# Anti-Xa level monitoring of low-molecular-weight heparin during intermittent venovenous hemofiltration

**DOI:** 10.1007/s00277-023-05290-7

**Published:** 2023-07-03

**Authors:** Lengnan Xu, Ying Sun, Songlan Wang, Chuanbao Li, Yonghui Mao

**Affiliations:** 1 Department of Nephrology, Beijing Hospital, National Center of Gerontology, Institute of Geriatric Medicine, Chineses Academy of Medical Sciences, Beijing, People’s Republic of China; 2 Department of Laboratory Medicine, Beijing Hospital, National Center of Gerontology, Institute of Geriatric Medicine, Chinese Academy of Medical Sciences, NO. 1 DaHua Road, Dong Dan, Beijing, 100730 People’s Republic of China

**Keywords:** Heparin, Low-molecular-weight, The coagulation grade, Intermittent venovenous hemofiltration therapy, Anti-factor Xa activity, Prospective observational study

## Abstract

Low-molecular-weight heparin (LMWH) is an anticoagulant used to prevent clotting during blood purification treatments. This study aimed to evaluate the clinical use of the anti-factor Xa level (anti-Xa) for monitoring LMWH anticoagulant levels during intermittent venovenous hemofiltration (IVVHF). This prospective observational study enrolled patients who required IVVHF for renal failure in Beijing Hospital between May 2019 and February 2021. The LMWH anticoagulation was assessed by the coagulation grade of the filter and line. One hundred and ten participants were included. There were 90 patients with a filter and line coagulation grade of ≤ 1 and 20 patients with grade > 1. The anti-Xa level of 0.2 IU/mL was a critical value. The multivariable logistic regression analysis showed that anti-Xa level > 0.2 IU/mL (odd ratio [OR] = 2.263; 95% CI: 1.290–4.871, *P* = 0.034) and cardiovascular disease (OR = 10.028; 95% CI: 1.204–83.488; *P* = 0.033) were independently associated with the coagulation grade of the filter and line. Anti-Xa level could monitor LMWH anticoagulation during IVVHF.

## Introduction

Continuous renal replacement therapy (CRRT) is an important component of the management of critically ill patients [1–3]. In CRRT, fluid is removed in a slow, controlled fashion, resulting in the maintenance of solute, acid-base, and electrolyte balance [1–3]. During CRRT, there is continuous contact between the circulating blood and extracorporeal circulation tubing. Inadequate anticoagulation often leads to line clotting, while excessive anticoagulation increases the risk of bleeding, cardiovascular events, and mortality [4, 5]. Therefore, individualized and precise anticoagulation protocols must be designed to improve CRRT outcomes and patient survival.

Intermittent venovenous hemofiltration (IVVHF) is the most used modality for CRRT [1–3]. Citrate anticoagulation is recommended as the first-line regimen for CRRT [6, 7]. On the other hand, heparin use remains common in China because of the difficulty in obtaining citrate and the lack of experience in its application in many centers.

Many investigators compared the efficacy of low-molecular-weight heparin (LMWH) and unfractionated heparin [8–10]. LMWH seems to be safer than unfractionated heparin: due to the complex pharmacokinetics of unfractionated heparin, its effects and side effects are unpredictable, and there is a high risk of bleeding and even life-threatening complications such as heparin-induced thrombocytopenia (HIT). LMWH has been shown to significantly reduce the incidence of HIT[11]. Most international hemodialysis guidelines still consider both forms of heparin to be equal as first-line anticoagulants [12, 13], while the European Best Practice Guidelines recommend LMWH in preference to unfractionated heparin [14]. LMWH is used in clinical practice because of its convenience and safety. At present, the most commonly used LMWHs include enoxaparin and LMWH sodium. These agents have different chemical structures, pharmacokinetics, and anticoagulant activities [15].

LMWH dose is usually adjusted based on clotting of the dialyzer or the occurrence of bleeding or adverse events. This approach is not objective or accurate. Nephrologists gradually realized that anti-factor Xa activity (anti-Xa) level could be used to monitor the degree of anticoagulation induced by LMWH [16–18]. Still, there are no reliable data to guide the interpretation of anti-Xa level during IVVHF. In order to monitor or appropriately adjust the LMWH dose, an accurate interpretation must be made of the anti-Xa level.

Therefore, this study aimed to evaluate the clinical use of the anti-Xa for monitoring LMWH anticoagulant levels during IVVHF.

## Methods

### Study design and participants

This prospective observational study enrolled patients who required IVVHF treatment for renal failure of various etiologies in Beijing Hospital between May 2019 and February 2021. We screened every patient with renal failure according to the order of visiting the hospital.

The inclusion criteria were (1) ≥ 18 years of age, (2) required IVVHF due to renal failure of various etiologies, and (3) planned use of anticoagulants. The exclusion criteria were (1) incomplete clinical data; (2) inappropriate application of heparin-based anticoagulants such as during acute bleeding, thrombotic or embolic events that required additional anticoagulants, blood platelet < 100×10^12^/L and coagulation dysfunction, or hepatic failure; or (3) patients did not agree to participate in this study.

The Ethics Committee of Beijing Hospital approved the study (2019BJYYEC-065-02). All study participants provided written informed consent.

### Procedures

The clinical data were obtained from the medical records, including sex, age, weight, medications (aspirin, clopidogrel), complications, blood coagulation status of the pipeline, bleeding events after the end of treatment, and serum creatinine. At the beginning of IVVHF treatment, a single LMWH bolus was injected intravenously, and it was not repeated afterwards. LMWH anticoagulation was given empirically based on patient weight (mainly according to 60–80 IU/kg) [19], and drug selection was based on the patient’s condition. LMWHs include LMWH sodium (0.2 mL: 2500 AXaIU, Qilu) or enoxaparin (0.4 mL: 4000 AxaIU; 0.6 mL: 6000 AxaIU, Sanofi Synthelabo France). LMWH sodium was applied for emergency room patients, and enoxaparin was applied for other patients according to access to the drugs. The treatment time of IVVH was 6 h, and blood was drawn at the end of treatment. First, the ultrafiltration rate was set to 0, and then, the blood flow rate was slowed to 100 mL/min. After 15–30 s, the blood samples were extracted from the arterial end (the tubing from the patient’s blood access site to the blood inlet of the dialyzer or filter). Anti-Xa levels were detected within 30 min. After blood centrifugation (3000 rpm, 5 min), the chromogenic substrate method (HemosIL, Liquid Anti-Xa, liquid chromogenic substrate S-2732, ACL TOP 750, Hartwell Road, Bedford, MA, USA) was used to detect the anti-Xa level.

The primary end point was defined as the coagulation grade of the filter (evaluated by two experienced blood purification nurses); the secondary end point was bleeding events (evaluated by two experienced blood purification specialists). The coagulation grade of the filter and lines of LMWHs was measured at 6 h of IVVHF and collected. The coagulation grade of the filter and lines included four grades, 0 was no residual blood and clots; 1 was residual blood occupies about 1/3 of the filter area, and a clot occurs in one end of the arterial or venous pot; 2 was residual blood occupies more than 1/2 of the area of the filter, but it is not completely blocked, and blood clots occur at both ends of the arterial or venous pot; 4 was the entire filter is blocked by residual blood, and blood clots fill the arterial or venous pot [20–22] (Table 1). Major bleeding was defined as life-threatening or death due to bleeding from vital organs. Minor bleeding was defined as other conditions such as subcutaneous bleeding.

**Table 1:** The coagulation grade of the filter and lines

Grades	Filter	Lines
0	No residual blood and clots	No residual blood and clots
1	Residual blood occupies about 1/3 of the filter area	A clot occurs in one end of the arterial or venous pot
2	Residual blood occupies more than 1/2 of the area of the filter, but it is not completely blocked	Blood clots occur at both ends of the arterial or venous pot
3	The entire filter is blocked by residual blood	Blood clots fill the arterial or venous pot

### Statistical analysis

Statistical analysis was performed using SPSS 23.0 (IBM Corp., Armonk, NY, USA). The data that conformed to a normal distribution were presented as means and standard deviations (SD) and analyzed using Student’s *t*-test. Continuous variables with a non-normal distribution were presented as medians (quartiles) and compared with the Mann-Whitney *U*-test. Categorical variables were presented as *n* (%) and analyzed using the chi-square test or Fisher’s exact test. Univariable and multivariable logistic regression analyses were conducted to examine the association between anti-Xa level and the coagulation grade of the filter and lines. A two-tailed *P*<0.05 was considered statistically significant.

## Results

Two hundred and three participants were recruited, but 93 were excluded due to non-heparin drugs (e.g., citrate) being used, and 110 were finally included in this analysis. Ninety participants (81.81%) had filter coagulation grade ≤ 1 and 20 patients (18.2%) had filter coagulation grade > 1. Participants with coagulation grades >1 more often used aspirin (40.0% vs. 33.3%, *P* = 0.040) but less often clopidogrel (25.0% vs. 31.1%, *P* = 0.001) than the participants with the grades < 1. The proportions of participants with diabetic nephropathy (75.0% vs. 55.6%, *P* = 0.024) and cardiovascular disease (85.0% vs. 41.1%, *P* = 0.039) were significantly higher in participants with coagulation grades > 1 than in participants with coagulation grades < 1. The anti-Xa levels of participants with coagulation grades ≤ 1 were significantly higher than those with coagulation grades > 1 (0.26±0.21 IU/mL vs. 0.11±0.08 IU/mL, *P*<0.001) and decreased with the increase of coagulation grades (Tables 2 and 3). From Table 3, it was clear that the anti-Xa level of 0.2 was a critical value. If the anti-Xa level was above 0.2, there would be no clotting events, whereas if it is below 0.2, clotting may occur easily.

**Table 2:** Comparison of clinical characteristics between the different coagulation grades of the filter and line

Parameter	Coagulation grades of the filter and line		
	<1 (*n*=90)	>1 (*n*=20)	*P*
Male (%)	65 (72.22)	13 (65.00)	0.117
Age (years)^#^	69.17±13.21	69.50±15.51	0.937
Weight (kg)^#^	67.52±11.69	63.57±10.03	0.394
Combined medications			
Aspirin	30	8	0.040
Clopidogrel	28	5	0.001
Underlying disease			
Diabetic nephropathy	50	15	0.024
Hypertension	46	11	0.805
Cerebrovascular disease	18	14	0.372
Cardiovascular disease	37	17	0.039
Serum creatinine (μmol/L)^#^	539.85±259.12	471.17±270.31	0.396
LMWH (enoxaparin)	52	9	0.329
Dose, IU/kg			0.778
<60	27	7	
60–79	46	8	
≥80	18	4	
Anti-Xa (IU/mL)^#^	0.26±0.21	0.11±0.08	<0.001

^#^Data are expressed as mean ± standard deviation; *P*<0.05

**Table 3:** Anti-Xa of different coagulation levels

Filter coagulation level	0 (*n*=72)	1 (*n*=18)	2 (*n*=12)	3 (*n*=8)
Anti-Xa (IU/mL)^#^	0.28±0.19	0.24±0.23	0.12±0.10	0.09±0.08

^#^Data are expressed as mean ± standard deviation; *P*<0.05

Furthermore, the multivariable logistic regression analysis showed that anti-Xa level > 0.2 IU/mL (odd ratio [OR] = 2.263; 95% CI: 1.290–4.871, *P* = 0.034) and cardiovascular disease (OR = 10.028; 95% CI: 1.204–83.488; *P* = 0.033) were independently associated with the coagulation grade of the filter and line (Table 4).

**Table 4:** Multivariate logistic regression analysis for the coagulation grade of the filter and line

Characteristic	Patients	OR (95% CI)	*P*
Dose, IU/kg			0.576
Anti-Xa (IU/mL)			
≥0.2	71	2.263 (1.290–4.871)	0.034
<0.2	39		
Cardiovascular disease	54	10.028 (1.204–83.488)	0.033
Aspirin	38	11.814 (0.901–153.380)	0.303
Clopidogrel	33	0.190 (0.016–2.241)	0.766
Diabetic nephropathy	65	7.251 (0.735–56.197)	0.099

Throughout the study, we did not observe major bleeding events. There was no statistical difference in minor bleeding events at different Anti-Xa levels (*P* = 0.975) (Table 5).

**Table 5:** Bleeding events at different anti-Xa levels

Anti-Xa (IU/mL)	≥0.2 (*n*=71)	<0.2 (*n*=39)	*P*
Major bleeding	0	0	
Minor bleeding (%)	7 (9.86)	3 (7.69)	0.975

## Discussion

The results suggest that anti-Xa level > 0.2 IU/mL and cardiovascular disease were independently associated with the coagulation grade of the filter and line.

Of note, the use of LMWH is not standardized in China, resulting in an important bias in this study and a decreased generalizability. Hence, in this study, LMWH was prescribed according to clinical experience and based on body weight, and 81.9% of the patients achieved an acceptable filter coagulation grade at the end of hemodialysis. LMWH is cleared primarily by renal metabolism, and its half-life is prolonged by impaired renal function, leading to LMWH accumulation [23]. Accumulating LMWH causes a progressively greater inhibition of factor Xa, increasing the susceptibility to bleeding [24]. During CRRT, LMWH is partially eliminated by the filter [25]. The monitoring and adjustment of LMWH for CRRT with patients who have renal failure should be carefully considered [26]. Tsang et al. [27] showed that enoxaparin at 1.5 mg/kg/24 h (6.25 IU/kg/h) was effective for anticoagulation in patients with CRRT and had systemic anticoagulant effects. In the present study, enoxaparin dosage was 6.81±4.37 IU/kg/h, and LMWH sodium dosage was 5.49±3.45 IU/kg/h; both regimens were at doses greater than reported in the literature [28]. At this dose, some patients still experienced severe coagulation in the filter at discharge time. Thus, it is important to monitor anti-Xa during the use of LMWH.

Anti-Xa is used clinically mainly for LMWH dose monitoring [29]. The present study illustrated that anti-Xa was highly associated with filter coagulation grade, with low anti-Xa being more likely to lead to coagulation. Final filter coagulation grade was independently associated with anti-Xa and cardiovascular diseases and not with body weight, blood creatinine level, or multiple drug combinations. Low anti-Xa level means that the risk of coagulation in the patient’s filter was increased; thus, clinicians should increase the anticoagulant dose appropriately.

In a randomized controlled study, Becker et al. [30] found that patients with significant renal impairment had greater fluctuations in anti-Xa level, and more patients experienced major bleeding events than patients without renal impairment. Therefore, there is a need to identify a range of anti-Xa that is effective in hemodialysis patients while avoiding drug overdose. Unfortunately, there are no uniform standards [31]. Laposata et al. [32] reported that peak and trough level of anti-Xa correlated well with the efficacy and safety of LMWH. Most extracorporeal life support organizations use anti-Xa activity measurements as part of their anticoagulant protocols, with a target level of 0.3–0.7 IU/mL [28]. Still, the present study suggested, for the first time, that the optimal cutoff value for anti-Xa at 6 h of IVVHF was 0.20 IU/mL.

Occasionally, the results of anti-Xa to assess LMWH fail to correspond exactly to the degree of bleeding and clotting. This failure might be because the samples for anti-Xa are plasma that lacks platelets; thus, the tests ignore the effects of platelets, the cell surface, and thrombin production on bleeding and clotting [33]. The authors are performing additional studies using anti-Xa and thromboelastography (TEG) to assess the status of bleeding coagulation in patients with renal failure.

We found that anti-Xa and cardiovascular disease are protective against filter and line occlusion by logistic regression. Anti-Xa higher than 0.2 IU/mL will obviously significantly reduce the blood coagulation and will not additionally increase the risk of bleeding (based on the data, it appears that bleeding events do not show a statistically significant difference when the value is greater than 0.2 IU/mL). The role of CAD in coagulation events was currently not well understood. It might be related to the use of antiplatelet aggregation, but our analysis, limited by a small sample size, did not find any association with aspirin and clopidogrel. In addition, there are some statistical issues. Because of the small sample size, only three no CAD patients had blood clotting, which resulted in “extremely small cells” in the relationship between variables and outcomes. This situation can easily lead to a wide OR value. Therefore, future larger-scale studies are needed for further analysis. We will continue to observe this phenomenon.

This study has several limitations. First, even though it was a prospective study, no power analysis was performed, and there were no control groups. Second, the sample size was relatively small, and the patients were conveniently included. Only anti-Xa levels were measured, and a complete coagulation marker panel was not assessed. Additionally, there was one more thing that must be mentioned: the anti-Xa level measurement was not standardized, and it could vary according to different commercial assays.

In conclusion, anti-Xa level might be used to monitor the anticoagulation of LMWH during IVVHF. Future multicenter studies with larger sample sizes will be essential to validate the results.

## Data Availability

All data generated or analyzed during this study are included in this article.
